# Effect of an intraoperative periradicular application of platelet-rich fibrin (PRF) on residual post-surgical neuropathic pain after disc herniation surgery: study protocol for NeuroPRF, a randomized controlled trial

**DOI:** 10.1186/s13063-023-07420-y

**Published:** 2023-06-19

**Authors:** Julien Todeschi, Guillaume Dannhoff, Andres Hugo Coca, Daniel Ionut Timbolschi, François Proust, François Lefebvre, Vincent Lelievre, Pierrick Poisbeau, Laurent Vallat, Eric Salvat, Yohann Bohren

**Affiliations:** 1 Service de Neurochirurgie, Hôpitaux Universitaires de Strasbourg, Strasbourg, France; 2 Centre d’Evaluation Et Traitement de La Douleur (CETD), Hôpitaux Universitaires de Strasbourg, 1 Avenue Molière, 67200 Strasbourg, France; 3 Faculté de Médecine, Université de Strasbourg, Strasbourg, France; 4 Service de Santé Publique, Hôpitaux Universitaires de Strasbourg, Strasbourg, France; 5 Centre National de La Recherche Scientifique, Institut Des Neurosciences Cellulaires Et Intégratives, Strasbourg, France; 6 Département de Génétique Moléculaire Des Cancers, Pôle de Biologie, Hôpitaux Universitaires de Strasbourg, Strasbourg, France

## Abstract

**Background:**

The prevalence of post-surgical lumbar neuropathic radiculopathy is approximately 30%. Poor response to the recommended treatments for neuropathic pain, namely antidepressants and/or gabapentinoids, requires the development of new techniques to prevent chronic pain. One such well-tolerated technique is the administration of autologous plasma enriched in platelets and fibrin (PRF). This approach is largely used in regenerative medicine owing to the anti-inflammatory and analgesic properties of PRF. It could also be an interesting adjuvant to surgery, as it reduces neurogenic inflammation and promotes nerve recovery, thereby reducing the incidence of residual postoperative chronic pain. The aim of the present study is to evaluate the benefit of periradicular intraoperative application of PRF on the residual postsurgical neuropathic pain after disc herniation surgery.

**Methods:**

A randomized, prospective, interventional, controlled, single-blind study with evaluation by a blind outcome assessor will be performed in Strasbourg University Hospital. We will compare a control group undergoing conventional surgery to an experimental group undergoing surgery and periradicular administration of PRF (30 patients in each arm). The primary outcome is the intensity of postoperative neuropathic radicular pain, measured by a visual analog scale (VAS) at 6 months post-surgery. The secondary outcomes are the characteristics of neuropathic pain (NPSI), the quality of life (SF-12 and PGIC), the presence of anxiety/depression symptoms (HAD), and the consumption of analgesics. We will also carry out transcriptomic analysis of a panel of pro- and anti-inflammatory cytokines in blood samples, before surgery and at 6 months follow-up. These gene expression results will be correlated with clinical data, in particular, with the apparition of postoperative neuropathic pain.

**Discussion:**

This study is the first randomized controlled trial to assess the efficacy of PRF in the prevention of neuropathic pain following surgery for herniated disc. This study addresses not only a clinical question but will also provide information on the physiopathological mechanisms of neuropathic pain.

**Trial registration:**

This study is registered at ClinicalTrials.gov: NCT05196503, February 24, 2022.

**Supplementary Information:**

The online version contains supplementary material available at 10.1186/s13063-023-07420-y.

## Background

Neuropathic pain is defined by the International Association for the Study of Pain (IASP) as “pain resulting from an injury or disease affecting the somatosensory system” [1]. From an epidemiological point of view, several large surveys using screening tools have been carried out in different countries. The STOPNEP study carried out in France on more than 30,000 individuals made it possible to estimate the prevalence of chronic pain at approximately 30%, including 7% with neuropathic characteristics, and similar results were yielded in a French 2018 study by Chenaf et al. [2, 3]. In other countries, similar estimates of prevalence (i.e., 6–10%) have been reported [4]. Among the most cited causes is a disc herniation, in almost 1 out of 5 cases [5]. Neuropathic pain remains underdiagnosed in general, with a significant delay in treatment, leading to excessive consumption of opiates and medical nomadism [6]. Thus, the intensity, persistence and impact of chronic pain significantly affect quality of life [7].

Acute lower back pain is one of the most commonly observed acute pains in clinical practice. Its natural history is most often favorable, with healing usually occurring within a few weeks. The problem appears when nerve root pain occurring in the lower back as a result of discoradicular conflict persists. Among these patients, some will benefit from spinal surgery. However, after spinal surgery, some patients will develop residual chronic pain, which has been shown to occur in 10–50% of cases [8]. For approximately 50–70% of these cases, we note the persistence of neuropathic symptomatology [9].

From a pathophysiological point of view, disc compression on the root quickly induces intraneural edema, leading to nerve fiber degeneration complications a few months later [10]. Similarly, intervention with surgical instruments may leave traces, such as inflammatory changes in the dura mater, thus modifying the volume of the thecal sac, or fibrotic scarring at the operating site, which can induce spatial and therefore mechanical stresses. In addition, an alteration of the perineural vascularization, particularly in the vasa nervorum, has been demonstrated in lumbar radiculopathy induced by chronic mechanical compression [11]. This ischemic theory postulates that compression causes venous stasis and/or a decrease in arterial flow within nerve root vascularization, inducing cellular hypoxia and nerve degeneration, thus leading to neuroinflammation phenomena of the nerve roots. Dorsal root ganglion (DRG) cells are involved in the pathophysiological mechanisms of initiation and maintenance of neuropathic pain [12]. Ultimately, the pathophysiology seems to be based on ubiquitous phenomena: a compression or mechanical conflict complicated by ischemic events, followed by degeneration of the nerve fibers, all associated with mechanisms of neuroinflammation [13].

The development of numerous animal models of neuropathic pain has made it possible to clarify some of the pathophysiological mechanisms. Communication between the nervous system and the immune system, in particular via cytokines at the peripheral or central level, contributes to the development and maintenance of neuropathic pain [14]. This neuro-immune balance has been the subject of much research in recent years [15]. Indeed, following a neurological lesion, the activation of immune and glial cells within the nervous tissue, at the site of the injured nerve, the DRG, then the neuraxis, leads to a release of cytokines, initiating and maintaining neuropathic pain [16–18]. Clinical studies have also shown neuropathic pain to induce changes in cytokine expression in nerve structures, such as the nerve and DRG, but also in cerebrospinal fluid [18]. As such, direct or indirect cytokinergic modulation could prove to be an effective therapeutic option against neuropathic pain.

Growth factors such as platelet-derived growth factor (PDGF) have been shown to decrease pro-inflammatory cytokine synthesis [19, 20]. Platelets, in addition to their well-known hemostasis role, contain a large number of growth factors (PDGF, transforming growth factor (TGF-β), etc.) that could play a role in this pro/anti-inflammatory balance [21]. A technique for concentrating growth factors contained in platelets allows to obtain platelet-rich plasma (PRP) by centrifugation of whole blood, then removal of the fraction containing large quantities of blood platelets. PRP injection is an autologous therapeutic tool that has recently emerged. It presents interesting nerve regeneration and analgesic capacities [18].

Other studies go even further by adjusting centrifugation speeds to successfully produce plasma rich in platelets and fibrin (PRF) [22]. Thanks to its fibrin network, PRF has the particularity to solidify within a few minutes after injection. This allows to limit the diffusion space, thereby resulting in an increase in the growth factors local concentration. This technique also allows gradual release (> 1 month) as compared to PRP injection alone [23].

In light of these properties, PRF would be interesting to use in the prevention of neuropathic pain, in particular within the context of spinal surgery. In the current protocol, we have chosen to focus on the prevention of residual neuropathic pain following surgery for herniated disc. Although this study population does not represent the majority of postoperative pain following spinal surgery, a selective population is required in order to assess the effectiveness of PRF in preventing neuropathic pain. To date, this study is the only randomized controlled trial designed to address this question.

## Methods and analysis

### Study design

The present NeuroPRF trial is designed as a superiority, prospective, single-center, randomized, parallel group study, conducted in a single-blind manner, with evaluation by a blind outcome assessor. Eligible patients will be randomized into one of two treatment groups: experimental group (surgery and periradicular administration of PRF) or control group (reference treatment, surgery alone). Patients will be followed for a total of 6 months. This manuscript used the SPIRIT reporting guidelines.

### Involvement in the design of the protocol

Patients and the public were not involved in the design of this study.

### Aim and objectives

The aim of the NeuroPRF trial is to determine whether the intraoperative periradicular application of PRF can prevent residual post-surgical neuropathic pain after disc herniation surgery.

The primary objective is to evaluate the effect of an intraoperative periradicular application of PRF on the intensity of postoperative residual leg neuropathic pain 6 months after primary surgery for a herniated disc.

Secondary objectives evaluate:

1. The incidence and characteristics of residual neuropathic pain;
2. Quality of life;
3. Anxious and depressive symptoms;
4. The correlation between neuropathic pain and gene expression of a panel of cytokines in the blood (specific search for a panel of pro- and anti-inflammatory cytokines);
5. The use of analgesics;
6. Safety.

### Outcomes

Study outcomes are assessed by a designated, blinded study investigator, and the patient (self-assessment), who is also blind to their treatment arm. All outcomes will be assessed at baseline (preoperatively) and at 2 weeks and 6 months after the surgery.

The primary outcome is the intensity of postoperative neuropathic radicular pain, measured by a visual analog scale (VAS). The neuropathic radicular pain will be confirmed by a clinical examination and a “Douleur Neuropathique 4” (DN4) score ≥ 4. Thus, VAS for leg pain will be measured during all follow-up visits: baseline (preoperative), 2 weeks and 6 months postoperative. VAS leg pain will be scored on a 0 to 10 points scale with increments of 1 point (0 being zero pain and 10 being the maximum pain possible).

In order to assess the presence and the neuropathic pain characteristics, the quality of life and the anxiety/depression syndromes, secondary outcomes will be measured at baseline (preoperative), 2 weeks and 6 months postoperative:

1. The incidence and characteristics of residual neuropathic pain, measured by the DN4 and the Neuropathic Pain Symptom Inventory (NPSI) questionnaires, respectively;
2. The evolution of quality of life with the 12-item Short Form Survey (SF-12) and Patients’ Global Impression of Change (PGIC) questionnaires;
3. Anxiety and depressive symptoms with the Hospital Anxiety and Depression (HAD) questionnaire;
4. The level of gene expression of a panel of pro- and anti-inflammatory cytokines in the blood (Table 1) using multiplex quantitative PCR;
5. The use of analgesics;
6. Incidence of adverse events (AE) and serious adverse events (SAE).

**Table 1:** Panel of genes used for gene expression analysis

**Pro-inflammatory cytokines**	TNF-α, IL-1β, IL-6, IL-8, IL-17, IL-18, MCP-1, RANTES, CXCL2, fractalkine
**Anti-inflammatory cytokines**	IL-2, IL-4, IL-10, IL-1RA
**Signal transduction proteins**	IκB, NFκB p65 subunit

*TNF* tumor necrosis factor, *IL* interleukin, *MCP* monocyte chemoattractant protein, *RANTES* regulated on activation normal T cell expressed and secreted, *CXCL2* chemokine (C-X-C motif) ligand 2, *IL-1RA* interleukin 1 receptor antagonist, *NFκB* nuclear factor kappa B, *IκB* inhibitor of nuclear factor kappa B

### Setting and patient recruitment

The study will take place at the Pain Center and the Department of Neurosurgery of Strasbourg University Hospital, in collaboration with researchers from the Institute of Cellular and Integrative Neuroscience (INCI) at the University of Strasbourg. Coordination and monitoring of the study will be carried out by the investigating team.

Patients will be recruited by the consulting neurosurgeon in the Department of Neurosurgery at Strasbourg University Hospital. Patients consulting for leg pain due to a disc herniation, for whom surgical intervention is indicated, will be referred for participation in this clinical study. During this routine consultation, a screening interview will be conducted for each potential participant using a questionnaire verifying eligibility criteria.

After verification of eligibility criteria, written information about the study will be provided to the patients, who will then be able to reflect on their decision to participate or not for approximately one week. Patients will be re-contacted by the study investigator, and, if they consent to participate, will attend an enrolment visit. After a final verification of eligibility, the patient will provide written informed consent.

In the consent form, participants will be asked if they agree to the use of their data, should they choose to withdraw from the trial. Participants will also be asked for permission for the research team to share relevant data with the Universities taking part in the research, or with regulatory authorities, where applicable. This trial does not involve collecting biological specimens for storage.

To improve adherence to intervention protocols, the neurosurgeon during the selection visit and the investigating physician during the inclusion visit will explain to the patient the expected benefits, in particular the reduction of neurogenic inflammation of the nerve root, the improvement of nerve healing and thus the potential prevention of chronic postoperative pain. To improve monitoring adherence, such as blood draws, they are taken with the patient's consent as part of the standard of care process. There are no additional visits related to the protocol.

The inclusion criteria are:

- Patient > 18 years old;
- Patient with a diagnosis of radiculopathy on lumbar disc herniation and for whom surgery has been scheduled in the Department of Neurosurgery;
- Patient affiliated to a social security health insurance scheme;
- Patient able to understand the objectives and risks of research and to give informed, dated and signed consent;
- Negative blood pregnancy test for female participants of reproductive age recorded at the inclusion visit and effective contraception used throughout the study.

The exclusion criteria are:

- Patient with a history of lumbar spinal surgery (multiple herniated discs, herniated disc other than lumbar);
- Patient with HIV, active cancer, HBV, HCV;
- Patient receiving long-term systemic corticosteroid therapy;
- Patient with an American Society of Anesthesiology (ASA) score > 3 during the routine pre-surgery consultation with the anesthetist;
- Inability to give the patient informed information (patient in an emergency or life-threatening situation, difficulties in understanding);
- Patient in exclusion period (determined by a previous or ongoing study);
- Subject under safeguard of justice;
- Subject under curatorship;
- Patient actively breastfeeding.

### Timeline and description of processes

An initial selection of patients will be carried out during a routine consultation with the practicing neurosurgeon, 30 days (± 20 days) prior to surgery. During this visit, the patient will be provided with the necessary study information, the eligibility criteria will be verified, and a clinical examination will be carried out. If the patient is eligible and decides to participate, an enrolment visit will be carried out 1 week (± 6 days) prior to surgery. At this visit, which takes place at the pain center (Strasbourg University Hospital), written informed consent is provided obtained from the participant, who will then complete baseline questionnaires and undergo a clinical examination. Randomization is also carried out at this visit, by using web-based randomization software (CleanWEB® clinical trial management platform). The details of randomization will remain unavailable to the medical examinator and the participant during the study. Randomization will be carried out using an allocation ratio of 1:1, which will determine the treatment arm of the patient: the interventional group (surgery and PRF application) or the control group (surgery alone). The allocation sequence is generated by the statistician of the study, using computer-generated random numbers. There will be no special criteria for discontinuing or modifying allocated interventions. To reduce predictability of a random sequence, the randomization will be carried out with two randomly distributed block sizes. Randomization will be carried out without factors of stratification. A specified investigator will carry out the enrolment visits and randomization process. This investigator and the surgeons carrying out the intervention will be the only investigators not blind to participants’ treatment arm. Blinding of the evaluating investigators will remain in place until statistical analysis of the study results has been finalized. Equally, participants are blinded to the intervention that they have received (experimental or control).

**Fig. 1:**
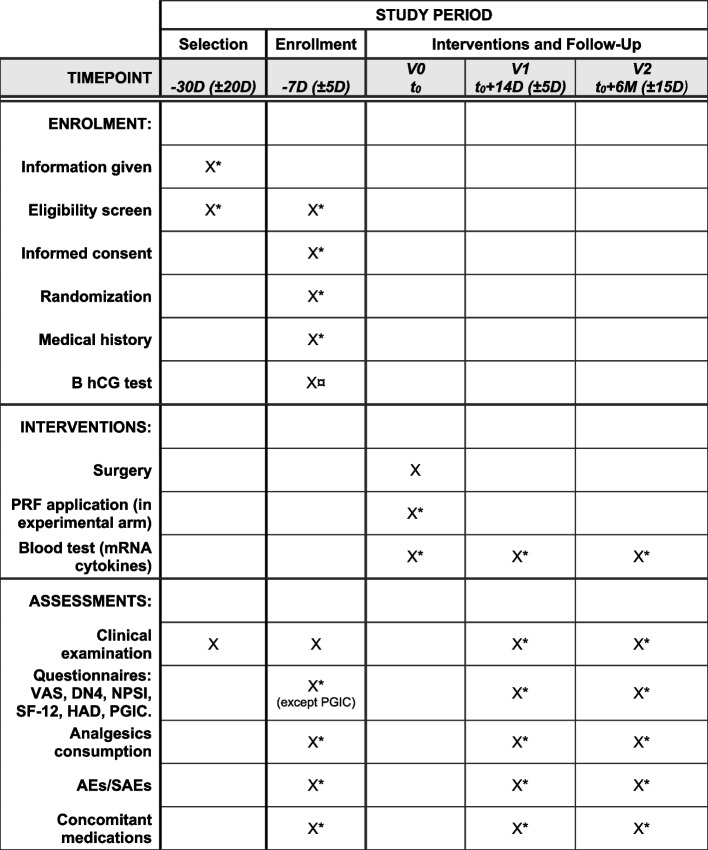
Schematic schedule of the NeuroPRF study. Asterisk symbol (*) indicates the following: variables carried out specifically for research purposes, outside of routine care; currency sign (¤) indicates the following: β-hCG test is carried out at a routine anesthetist consultation; D, days; M, months; AE, adverse event; SAE, serious adverse event; VAS, visual analog scale; DN4, Douleur Neuropathique 4; NPSI, Neuropathic Pain Symptom Inventory; SF-12, 12-item Short Form Survey; HAD, Hospital Anxiety and Depression

The day of surgery is designated as V0. At V0, a blood sample will also be collected for RNA extraction and genes of interest expression analysis, just before the surgery. This will be considered as the basal condition. The participant will be examined at two further visits: 2 weeks (± 5 days) post-surgery (V1) and 6 months (± 15 days) post-surgery (V2). These visits will consist of a clinical examination, a blood sample collection for gene expression analysis, and completion of questionnaires. Figure 1 shows a schematic chart of the study timeline.

### Description of clinical examination and questionnaires

The baseline clinical examination carried out at the enrolment visit includes questions about pain descriptors and history. Medical history, comorbidities, and medications will also be documented. The clinical examinations carried out at V1 and V2 consist of a physical neurological examination including myotomal strength, reflex, and sensory testing (soft touch, pinprick, allodynia or hyperalgesia detection).

Participants will be asked to complete the following questionnaires:

- DN4 [24];
- NPSI [25];
- HAD [26];
- SF-12 [27];
- VAS for current leg pain [28];
- PGIC scale [29].

All are self-questionnaires, except for DN4, which will be carried out by the examining clinician. All questionnaires will be completed pre-operatively, at 2 weeks, and at 6 months post-operatively, with the exception of the PGIC scale, which will be completed only after the surgery (i.e., 2 weeks and 6 months post-operatively). Data from these examinations will be entered into the CleanWEB® e-CRF by the examining investigator.

### Description of interventions

Surgery will be carried out under general anesthesia for both groups. Surgical treatment of lumbar disc herniation relies on relieving the compression of the nerve root by the herniated disc. Prior to surgical incision, a blood sample of 5 mL will be taken for gene expression analysis. This must be carried out prior to surgery in order to avoid any systemic inflammatory phenomena caused by surgery itself.

*- Control group (surgery alone)*: The patient is placed in the knee-pectoral decubitus position under general anesthesia. The concerned intervertebral space is identified by radioscopy. The most common procedure implies a median posterior cutaneous incision, with subsequent detachment of the paravertebral muscles from the underlying vertebra. The interlaminar space is then approached through removal of the ligamentum flavum. Once the vertebral canal is accessed, epidural fat is retracted in order to visualize the dural sac and emerging spinal roots. Gentle traction on the dural sac and spinal roots allows exposure of the bulging disc at the compression point. The posterior longitudinal ligament is then incised and herniated disc removed with additional discectomy if necessary. Closure is finally performed with respect to each anatomical layer.

*- Interventional group (surgery and periradicular administration of PRF)*: The same surgical steps are carried out as those in the control group, with an additional periradicular administration of autologous PRF. Preparation of the PRF will be carried out during the surgery, in the operating theater. Twenty milliliters of venous blood will be collected from the perfusion device, in 2 sterile tubes without anticoagulant (Becton Dickinson‚ Vacutainer‚ 10 mL each), and immediately centrifuged at room temperature for 14 min at 1300 rpm in a suitable centrifuge (PRF duo, Process for PRF, Nice, France), according to the A-PRF protocol described by the manufacturer. Under the effect of coagulation activation and centrifugal forces, three layers will appear in the tube: a pellet of red blood cells at the bottom, an acellular plasma supernatant on the surface, and a PRF fibrin clot loaded with platelets in the middle. PRF in gel form is then recovered under sterile conditions by the neurosurgeon, who will shape it using tweezers so that it can be applied to the root lesion before layer-by-layer closing.

During the immediate postoperative period, all analgesics are permitted as per standard of care: paracetamol, NSAIDs, nefopam, tramadol, lidocaine, ketamine, morphine. For residual postoperative pain, level 1, 2, or 3 analgesics are permitted.

### Gene expression analysis

To study the mechanisms of initiation and maintenance of neuropathic pain, and in particular the involvement of the cytokinergic system [18], we will measure the expression of messenger RNA (mRNA) of selected pro-inflammatory and anti-inflammatory cytokines and signal transduction proteins (Table 1), extracted from peripheral blood mononuclear cells (PBMC). Indeed, the synthesis and release of cytokines are essentially ensured by glial and immune cells in neuropathic conditions [12]. These mononuclear immune cells therefore appear to be a good marker of cytokine involvement in the pathophysiological mechanisms behind the onset and maintenance of neuropathic pain [15].

A 5 mL sample of blood will be taken from participants at V0, V1, and V2 in a pseudonymized EDTA-anticoagulation LeucoSep vials (Greiner, ref.227288). Briefly, samples will be subjected to density gradient centrifugation (1000 × g, 10 min, 4 °C) to isolate PBMC. Collected PBMC will be washed (1000 × g, 10 min, 4 °C) in Hank’s buffer (Fisher Scientific, ref.24020–091), and red blood cells will be lyzed (Qiagen, ref.79217). After another wash (1000 × g, 10 min, 4 °C), two identical aliquots of highly-purified PBMCs will be collected; the first one will be resuspended in 600 μL of RNAlater Stabilization Solution (Invitrogen, ref.AM7020) and stored at – 20 °C for back-up. The second will be resuspended in 200 μL of 1-thiogycerol solution (Promega, ref.AS1340), vortexed and stored at – 20 °C for RNA extraction. Total RNA extraction will be performed on a Maxwell RSC device (Promega, ref.4500) according to the manufacturer’s instructions. Quality control will be performed on microfluidic chips prepared using the 6000 Nano Kit (Agilent) and loaded on the 2100 Bioanalyzer system (Agilent) according to the manufacturer’s instructions. RNA samples will be quantified on a Nanodrop 2000 spectrophotometer (Thermo Scientific). These high-quality, DNA-free RNA samples will then be stored at – 80 °C.

Four hundred nanograms of each RNA sample will be reverse-transcribed with the iScript gDNA Clear cDNA Synthesis Kit (Bio-Rad), which integrates an effective DNase digestion step to remove any trace contamination of genomic DNA.

Quantitative PCR will be performed using SYBR Green Supermix (Bio-Rad), on the iQ5 Real Time PCR System (Bio-Rad). Amplifications will be carried out in 42 cycles (20 s at 95 °C, 20 s at 60 °C, and 20 s at 72 °C). Primer sets for all genes of interest and housekeeping genes have been designed using Oligo6.0 and M-fold software. To ensure a high primer specificity and an optimal amplification efficacy for all primer sets in the given PCR conditions, we will use control samples to verify that amplification efficacy given by standard curves will be close to 100% (± 2%) and that amplification specificity, assessed by a melting curve study, will generate a single peak at the melting temperature expected by sequence analysis. Samples will be accurately dispensed in 96-well plates using a robotic workstation, for a final volume of 15 μL per well (Freedom EVO100; Tecan, Lyon, France). Targets will be amplified in triplicate, and threshold cycle (Ct) values will be determined. Fold changes in gene expression among groups will be calculated using the delta-delta Ct method. Gene expression data will be normalized to the arithmetic mean of housekeeping gene expression (ACTB, B2M, GAPDH and HPRT).

### Safety considerations

Adverse events are defined as any undesirable experience occurring to a subject during the study. All adverse events reported directly by the subject or observed by an investigator will be recorded. In the case of a suspected unexpected serious adverse reaction (SUSAR), the pharmacovigilance officer, in collaboration with the sponsor and investigators, will carry out the unblinding of the participant in question, via the CleanWEB® interface. There is no criterion or formal stopping rules. Indeed, the treatment is unique and, intraoperatively, there is no criterion for definitive discontinuation of the use of the experimental product as such. There is a very small risk that the treatment cannot be administered during surgery for logistical, medical or other reasons. Thus, the patient would then be withdrawn from the study.

No data monitoring committee was envisioned because the study was a low-risk intervention. However, the investigators agree to comply with the requirements of the sponsor and the competent authority for an audit or inspection of the study (Additional file 1). Consistency checks of the collected data will be performed by computer according to predefined rules between the sponsor and the investigator described in the monitoring plan. Requests for information or “queries” will be sent to the investigator to correct or clarify specific data. Any modification of data will be traced back via an audit trail that can be consulted with the CLEANWEB® software. A clinical research associate, delegated by the sponsor, will visit the study center at the beginning of the trial, then during the trial at the rate of once a year during the trial and at the end of the trial.

### Sample size determination

The sample size was determined by assuming that the mean of the VAS measures will be 5 ± 1 in the control group and 3 ± 1 in the intervention group. A sample size of 25 subjects per group will allow for a difference of at least 1 point between the two groups, with a power of 93.5% and a type I error of 5%. An additional 20% of subjects are added to take into account possible loss of follow-up, bringing the total to 30 subjects per group. Patients can withdraw from the study at any time, for any reason, and their decision to stop their participation will not affect their regular standard of care. Withdrawn participants will not be replaced. Patients that withdraw from the study will be considered as being lost to follow-up. There is no anticipated harm and compensation for trial participation. Moreover, there will be no provision for post-trial care. The patient will return to the usual standard of care pathway if needed.

A recruitment of 6–8 participants per month is necessary to achieve the required sample size. Given that the Department of Neurosurgery treat 3–5 patients per week for disc herniation surgery, sufficient recruitment should be feasible in the pre-defined timeframe. Moreover, the principal investigator will coordinate the follow-up and end-of-study visit with those scheduled with the surgeon to avoid loss of data.

### Statistical analysis

The analyses will be carried out using Bayesian methods.

Firstly, a descriptive analysis will be carried out. For the categorical variables, the frequency of each value and the cumulative frequency will be given. For the quantitative variables, location parameters (mean, median, minimum, maximum, first and third quartiles) and dispersion parameters (standard deviation, variance, range and interquartile range) will be given. The parameters will be estimated firstly on raw data, and secondly within the Bayesian paradigm, on the posterior distribution, for each variable.

Secondly, to compare the difference of the means of the VAS, a Bayesian linear regression will be performed. The regression will be simple linear or generalized depending on the distribution of the data. To meet the secondary objectives, Bayesian linear regressions will be performed for continuous variables, and Bayesian logistic regressions for categorical variables.

The regressions will be firstly carried out without adjustment and secondly with an adjustment on sex and age. Subgroup analyses are not planned.

The priors will be on the one hand very uninformative and on the other hand informative in the context of a sensitivity analysis.

For each analysis, the posterior distribution of the parameter of interest (rate, proportion, mean, odds ratio, regression coefficient, etc.) will be estimated using Markov chain Monte Carlo (MCMC) draws. The default number of iteration is 200,000 with a burning of 10,000 and a thinning of 2. Algorithm convergence will be assessed graphically and with the Gelman and Rubin’s convergence diagnostic. Autocorrelation will be assessed graphically and, if required, the number of iterations and thinning will be increased to reduce as much as possible the autocorrelation.

Credibility intervals will be calculated at 95% using the quantile method. A particular factor will be considered to exert an effect on the variable in question if the probability that the effect is greater than the reference value is greater than 0.975 or less than 0.025.

The proportion of missing data will be given for each variable. Missing data will be treated by simple deletion of cases if these values are very few or by multiple imputation in the opposite case.

Two different data sets will be defined:

- The intention-to-treat (ITT) data set, which contains all subjects who give their consent for the study and who undergo randomization, whether they follow or not the protocol properly (for instance if they do not follow the treatment or the visits planning).
- The per-protocol (PP) data set, which contains all subjects who, after randomization, undergo the treatment and who follow the protocol as planned.

The datasets analyzed during the current study and statistical code are available from the corresponding author on reasonable request, as is the full protocol.

## Discussion

The platelet-rich plasma was historically used in dentistry for its osteo-regenerative properties. This technique has been rapidly extended to other fields, such as rheumatology or sports medicine. In the field of pain, some studies have reported its analgesic properties on neuropathic pain of peripheral origin, while perineural injection of PRP has been shown to improve pain in patients with various etiologies, such as postherpetic neuralgia, chronic post-surgical pain, or traumatic peripheral nerve injury [30–32].

Furthermore, it could be an interesting adjuvant to surgery. In a case report, the use of intraoperative PRP during nerve decompression surgery following digital trauma was shown to significantly reduce neuropathic pain and improve functional recovery [33]. PRP could also be of interest for neurosensory recovery [34]. Following the example of these clinical studies, intraoperative PRP could act to reduce neurogenic inflammation, promote nerve healing, and thus reduce the incidence of residual postoperative chronic pain.

We elaborated the first randomized controlled trial to assess the efficacy of PRF in the prevention of neuropathic pain following surgery for herniated disc. This study not only addresses a clinical question but will also provide information on the physiopathological mechanisms of neuropathic pain. However, this trial has some limitations. This study is limited by its single-center design. The selected study population (participants undergoing spinal surgery for a herniated disc) can be considered a limitation in that this population does not represent the majority of patients who experience chronic pain following spinal surgery.

Thus, the interest of this therapy is that PRF could also be an interesting adjuvant to surgery and may thus block the initiation of neuropathic pain mechanisms [18].

## Trial status

This trial was registered at ClinicalTrials.gov and this article is based on the 2nd version of the protocol published in February 2022. It was not submitted earlier to be published due to changes in the study status. Recruitment of participants is intended to start in March 2022 and is expected to be completed no later than January 2025. The total duration of the study will be 3 years.

Any significant modification of the protocol (for example changes in eligibility criteria or analyses) will be the subject of a protocol amendment and will be transmitted to the concerned parties (sponsor, funder, principal investigator). If applicable, the protocol will be updated in the clinical trial registry.

## Supplementary Information

**Additional file 1.** The right of access to source data and documents.
