# Supplementary material for: Effect of an intraoperative periradicular application of platelet-rich fibrin (PRF) on residual post-surgical neuropathic pain after disc herniation surgery: study protocol for NeuroPRF, a randomized controlled trial
Source: Trials. 2023 Jun 19;24:418. doi: 10.1186/s13063-023-07420-y (PMC10280922; doi:10.1186/s13063-023-07420-y)
Supplement: Supplementary file 1 — Additional file 1. The right of access to source data and documents. [file 13063_2023_7420_MOESM1_ESM.docx]

**Additional file 1: the right of access to source data and documents**

The investigator authorizes direct access to the source data and documents in accordance with the legislation and regulation in place, to the persons in charge of the quality control of the research duly mandated for this purpose by the sponsor and to all persons called upon to collaborate in the trial. These persons take all necessary precautions to ensure the confidentiality of the information relating to the trial, the experimental products, the persons involved in the trial and in particular their identity and the results obtained. The data collected by these persons during quality control or audits should remain anonymous.

Investigators agree to comply with the requirements of the sponsor and the Competent Authority with respect to an audit or inspection of the study.

The audit may apply to all stages of the study, from the development of the protocol to the publication of the results and the classification of the data used or generated in the study.
